# Intravitreal bevacizumab improves trabeculectomy survival at 12 months: the bevacizumab in trabeculectomy study—a randomised clinical trial

**DOI:** 10.1136/bjo-2023-323526

**Published:** 2023-08-04

**Authors:** John A Landers, Sean Mullany, Jamie E Craig

**Affiliations:** Ophthalmology, Flinders University of South Australia, Adelaide, South Australia, Australia

**Keywords:** Glaucoma, Treatment Surgery

## Abstract

**Aims:**

To evaluate the effect of an intraoperative dose of intravitreal bevacizumab (Avastin) on surgical success following trabeculectomy with mitomycin-C (MMC) over 12 months.

**Methods:**

A single centre, parallel, double-blinded randomised, placebo-controlled trial recruiting patients requiring trabeculectomy for progressing glaucoma. Patients were randomised to intravitreal bevacizumab or placebo.

**Main outcome measure:**

The primary outcome of treatment success was defined by ‘complete success’ when intraocular pressure (IOP) remained less than a predefined target IOP without the requirement of topical medication, or ‘qualified success’ where topical medication was required to meet the predefined target IOP threshold. Secondary outcomes included the need for subsequent IOP-lowering interventions, and structural parameters associated with bleb function.

**Results:**

From 131 patients randomised to bevacizumab (n=65) or placebo (n=66), 128 patients completed 12 months of follow-up (98%). At 12 months, success rates were higher in the bevacizumab group (complete success: 94% vs 83%; p=0.015; qualified success: 98% vs 90%; p=0.033). Within the placebo group, the requirement for topical therapy was higher at 6 months (p=0.045) and 12 months (p=0.045), and the requirement for bleb needling was higher at 1 month (p=0.035). Blebs within the bevacizumab group were larger at 1 month (p<0.001) and demonstrated less vessel inflammation (p<0.0001).

**Conclusion:**

Bevacizumab given as a single intravitreal dose during trabeculectomy with MMC resulted in improved surgical success as 12 months. Furthermore, bevacizumab was associated with a significant reduction in the need for additional medication or further surgery to achieve target IOP. Bevacizumab was also associated with larger blebs that were less inflamed and required fewer subsequent interventions.

**Trial registration number:**

ACTRN12614000375651.

**WHAT IS ALREADY KNOWN ON THIS TOPIC**

- Trabeculectomy with adjuvant mitomycin-C (MMC) currently represents the gold standard surgical approach for progressing glaucoma. Adjuvant use of intraoperative anti-vascular endothelial growth factor (VEGF) agents has been suggested as a potential strategy to incrementally improve outcomes associated with this technique.

**WHAT THIS STUDY ADDS**

- This is the first double-blinded, randomised, placebo-controlled trial to investigate and demonstrate improved postoperative success rates through administration of a single dose of adjuvant bevacizumab (Avastin) in trabeculectomy+MMC surgery.

**HOW THIS STUDY MIGHT AFFECT RESEARCH, PRACTICE OR POLICY**

- The results of this study suggest that a single intraoperative dose of adjuvant intravitreal anti-VEGF may improve postoperative outcomes in individuals undergoing trabeculectomy+MMC for progressing glaucoma.

## Introduction

Trabeculectomy surgery has been the mainstay of surgical glaucoma management since its development in 1968.^1^ Although this technique has changed little over the last 50 years, there have been numerous attempts to improve success rates and lessen the risk of complications. Several incremental improvements were achieved through introduction of intraoperative antifibrotic agents, namely 5-fluorouracil (5FU) and mitomycin-C (MMC).^2 3^ Accordingly, trabeculectomy with MMC represents the current gold-standard surgical approach to medically refractory glaucoma.^4^ Despite improvements to surgical outcomes, there remains a proportion of patients who fail each year following surgery.

Healing following trabeculectomy surgery involves a fibroproliferative phase during the first week postoperatively, which may cause conjunctival fibrosis, leading to bleb failure.^5^ Vascular endothelial growth factor or VEGF is a cytokine, which has potent angiogenic and mitogenic actions, having a highly selective action on vascular endothelial cells, a secondary action on macrophage and fibroblast migration and modulating capillary vascular permeability.^6^ VEGF has been found to be present in higher amounts in the aqueous of glaucoma patients. It is also found in higher amounts in the tenon’s capsule of glaucoma patients undergoing trabeculectomy surgery. It modifies fibroblast activity, stimulating collagen cross-linking and contraction, resulting in scar formation.^7 8^ Higher VEGF levels in tenon’s tissue preoperatively are associated with a worse outcome following trabeculectomy surgery.^7^

Bevacizumab (Avastin) is a large humanised monoclonal IgG_1_ antibody whose main action is inhibition of VEGF.^9^ Bevacizumab was first developed in 2004 to treat colorectal cancer, but in 2005 was approved as an intravitreal injection for the management of macular oedema associated with age-related macular degeneration (AMD) and later retinal vein occlusion and diabetic retinopathy. VEGF has been implicated in the pathophysiology of various ocular conditions characterised by vascular proliferation (ie, AMD and rubeosis) or increased permeability (retinal vein occlusion and diabetic maculopathy).^10^ Treatment with anti-VEGF medications results in regression of neovascularisation and a reduction in vessel leakage.^10^ Bevacizumab used both in vitro and in vivo has been demonstrated to antagonise these actions of VEGF,^11^ with peak effect at 50 days,^12^ and biological effects which continue for up to 100 days following administration.^12^ Consequently, it has been proposed that bevacizumab may contribute to better outcomes following trabeculectomy surgery both in animal glaucoma models and in humans.^3 13 14^

Previous research has demonstrated that the most rapid period of postoperative trabeculectomy failure is during the first 2 months.^15^ We speculated that if fibroproliferation could be suppressed and failure could be prevented during this period, then trabeculectomy survival could be markedly prolonged. We hypothesised that a single adjunctive intraoperative intravitreal bevacizumab injection during trabeculectomy would inhibit this fibroproliferative phase of wound healing, reduce tenons and conjunctival thickening in the bleb and could lead to improved outcomes. The study was designed to investigate the effect of an adjunctive intraoperative intravitreal bevacizumab injection on trabeculectomy bleb function and morphology during the first postoperative 12 months, using a prospective randomised controlled double blinded trial design.

## Materials and methods

### Ethical approval and trial registration

The recruitment of study subjects conformed to the tenets of the Declaration of Helsinki. Ethical approval was obtained from the Southern Adelaide Clinical Human Research Ethics Committee (HREC; approval ID: 378.14) and authorisation was obtained from the Therapeutic Goods Administration to undertake a phase 4 clinical trial, which was registered at the Australian & New Zealand Clinical Trials Registry.

### Sampling criteria

Patients were recruited consecutively from glaucoma outpatient clinics at a single ophthalmic referral centre within a tertiary teaching hospital (Flinders Medical Centre, South Australia) and were classified as having glaucoma as defined as a progressive glaucomatous optic neuropathy with matching disc and visual field loss and requiring trabeculectomy surgery. In all instances, the indication for trabeculectomy surgery was progressing glaucoma as determined at the glaucoma surgeon’s clinical discretion. Patients were excluded if any of the following criteria were present: age less than 18 years old, unwilling or unable to give consent, unwilling to accept randomisation, unable to return for scheduled protocol visits, pregnant or breastfeeding women, active iris neovascularisation or active proliferative retinopathy, iridocorneal endothelial syndrome, glaucoma due to anterior segment dysgenesis or phacomatosis, aniridia or any need for glaucoma surgery combined with other ocular procedures except phacoemulsification cataract surgery (such as penetrating keratoplasty, vitrectomy surgery or anticipated need for such additional ocular surgery).

### Randomisation

Randomisation was conducted using a block permuted design, which was stratified for surgeon and for surgery type (primary trabeculectomy vs redo-trabeculectomy). Both surgeons and patients were masked to the allocation of the randomisation. Target intraocular pressure (IOP) was defined using methodology adopted from the Collaborative Initial Glaucoma Treatment Study (CIGTS) using the CIGTS VF score and the preoperative IOP.^16^ The CIGTS VF score is a method for grading the visual field from the Humphrey Field Analyzer 24–2 deviation plot, by calculating the number and depth of defects and scaling them to a range between 0 and 20.^16^ This score was then combined with the preoperative IOP ((1-(Preoperative IOP+CIGTS VF Score)/100)×Preoperative IOP) to give the target IOP.

### Interventions

Patients underwent trabeculectomy surgery in a standardised manner, using a fornix-based approach, a 3×5 mm scleral flap, a 0.5 mm sclerostomy, two fixed 10.0 nylon sutures and between two and four adjustable 10.0 nylon sutures. MMC 0.04% was used for 2 min for a low-risk case and for 3 min for a high-risk case. At the end of the operation, each patient received either intravitreal bevacizumab (Avastin; 1.25 mg in 0.05 mL) or balanced salt solution, as prepared by the hospital pharmacy. An intravitreal mode of administration was chosen over alternatives due to evidence that intravitreal anti-VEGF agents are anticipated to have prolonged biological effects.^12^

Postoperatively, topical chloramphenicol drops four times per day for 1 week and dexamethasone 1% drops 2 hourly for 1 week, then four times per day for 3 months were used.

### Monitoring

Patients were assessed postoperatively on day 1, week 1, week 4, week 8, week 12, week 26 and week 52. IOP and best central visual acuity (BCVA) were measured at each postoperative visit. Bleb photographs were performed on week 4, week 12, week 26 and week 52. The requirement for postoperative bleb needling with subconjunctival 5FU (5 mg) was performed at the surgeon’s discretion, in the presence of increasing IOP or bleb inflammation or fibrosis.

### Outcomes

Survival after trabeculectomy surgery was judged on functional and surgical grounds, which were based on the World Glaucoma Association guidelines on design and reporting of glaucoma surgical trials.^17^ Success on functional grounds was defined as ‘complete success’ where IOP remained ≤target IOP with no additional medication. ‘Qualified success’ occurred if IOP was ≤target IOP but included those who required additional topical medication to achieve this outcome. However, failure at this level occurred if IOP was >target IOP on at least two occasions despite maximal medical therapy, or if further glaucoma

filtration surgery was required. Failure on functional grounds occurred if the patient developed irreversible blindness in the operated eye as the result of glaucoma or the trabeculectomy under analysis (eg, suprachoroidal haemorrhage, phthisis and endophthalmitis),^17^ but not as the result of unrelated causes (eg, AMD and central retinal artery occlusion). Similarly, return to theatre in the early postoperative to address hypotonic maculopathy was not considered a determinant of treatment success as it constitutes an anticipated complication of glaucoma filtration surgery.

Success on surgical grounds was based on the morphology of the trabeculectomy bleb. The assessment of the bleb was performed using the Moorfields Bleb Grading Scheme.^18^ A single bleb grader (JAL), who was masked to the randomisation allocation, compared bleb extent (both central and peripheral), bleb height and also bleb vascularity with standardised photographs.

Based on the median outcome of previous work,^15 19–27^ the 1-year complete success rate for trabeculectomy surgery can be expected to be approximately 75%. We would expect that an improvement in success rate of 20% would be required for a change in practice to occur. We, therefore, required a complete 12 months of data from 100 patients (50 cases and 50 controls). This would allow us to detect a difference of at least 20% between groups, with 80% power and type 1 error of 5%. Anticipating as much as 20% loss to follow-up due to death and attrition, we aimed to sample a minimum of 125 patients. The SAS V.9.1 (SAS, Cary, North Carolina) was used for statistical analysis, including descriptive statistics, Student t-test and Cox proportional hazards model. Age and IOP at the time of surgery were used as continuous variables. Randomisation allocation, sex, surgeon, surgery type and combined trabeculectomy with phacoemulsification surgery were used as dichotomous variables. Test statistics, hazard ratios, 95% CIs, and p values are presented. p<0.05 was considered statistically significant.

## Results

**Figure 1:**
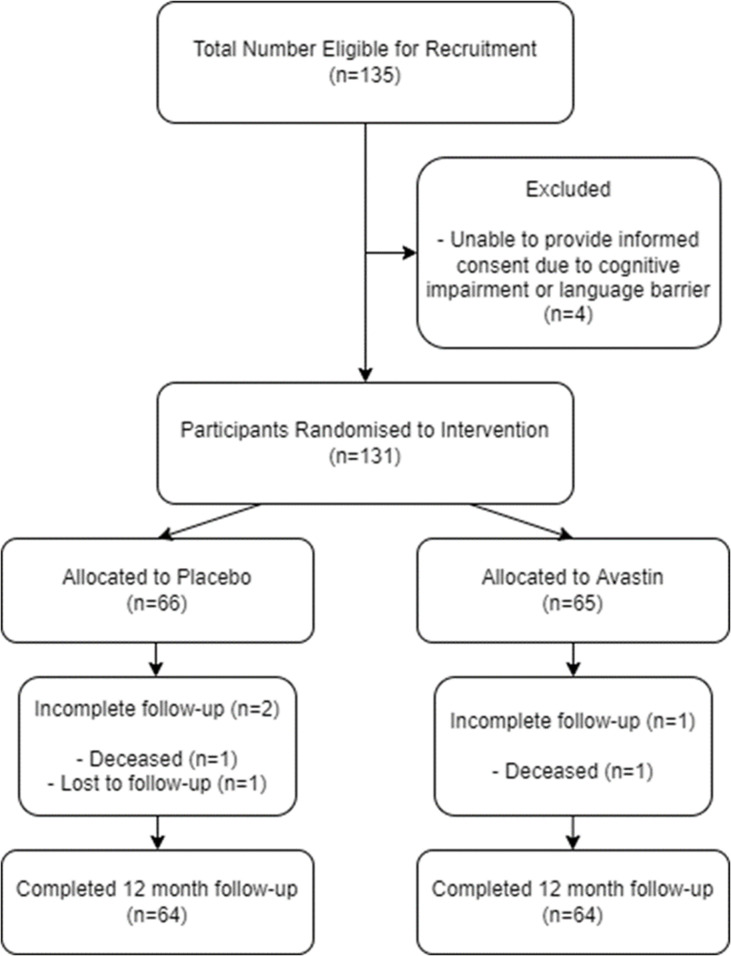
Patient recruitment flowchart.

Patients were recruited for this study between 20 July 2016 and 5 September 2018. We recruited 131 patients who were randomised to bevacizumab (n=65) or placebo (n=66). Cohort ethnicity was determined through self-reporting and included 129 individuals of European ancestry and two individuals of Asian ethnicity. There were no differences in age, gender, glaucoma type, glaucoma severity, risk of failure, number of preoperative medications, time since diagnosis or preoperative vision between randomisation groups (table 1). However, a higher proportion of patients randomised to receive bevacizumab underwent phacoemulsification cataract surgery at the time of trabeculectomy (22/65 (33.8%) vs 12/66 (18.2%); p=0.041). During the 12 months following trabeculectomy, one patient was lost to follow-up from the control (placebo) group and two patients died (one who received bevacizumab died from pulmonary fibrosis, and one who received placebo died from pneumonia). A total of 128 patients completed 12 months of follow-up (figure 1).

**Table 1:** Sample characteristics at baseline and 12 month follow-up

Parameter	Treatment group		P value
	**No bevacizumab**	**bevacizumab**	
**At baseline:**			
Sample size (n)	66	65	
Gender (male: female(% male))	28:38(42%)	37:28(57%)	0.10
Age (years±SD)	74±13	78±11	0.10
Baseline IOP (mm Hg)	20.9	21.3	0.80
Target IOP (mm Hg)	13.6	13.2	0.60
Glaucoma risk (n;(% high-risk))	34(51%)	32(48%)	0.79
Number of pre-operative topical medications (median (range))	3 (0–4)	3 (0–4)	0.74
Use of diamox pre-operatively (n(% of total))	14(21%)	12(18%)	0.69
Patients with ‘date of treatment’ onset information (n(% of total))	29(44%)	35(54%)	0.26
Duration of treatment (days; median (range))	4094(5611 845)	3677(6517 121)	0.42
Operation:			
primary trab: redo-trab (% primary)	59:7 (89%)	59:6 (91%)	0.79
Additional phaco (% of total)	12 (18%)	22 (33%)	0.04
Glaucoma phenotype:			
POAG	30	34	
NTG	8	10	
PACG	5	5	
PXG	7	11	
PDG	1	2	
Post-surgical	8	1	
Steroid induced	1	0	
Traumatic	1	1	
Uveitic	5	1	0.37
Best central visual acuity (ETDRS) (mean±SD)	0.37 (0.38)	0.37 (0.34)	0.99
Visual field score (median; IQR)	13 (8.5,15.5)	13 (8.5,15.0)	0.80
At 12 months:			
Deaths	1	1	0.99
Loss to follow-up*	0	1	*NA*
Completed 12 months follow-up	65	63	0.99

Independent sample paired t-tests and Chi-squared tests were performed to compare baseline and 12 month follow-up parameters between treatment groups. visual field scores that were non-parametrically distributed, were compared using a Mann-Whitney U test.

*One patient from the control group was lost to follow-up at 3 months.

ETDRS, early treatment diabetic retinopathy study; IQR, interquartile range; NA, not applicable; NTG, normal-tension glaucoma; PACG, primary angle-closure glaucoma; PDG, pigmentary dispersion glaucoma; phaco, phacoemulsification cataract surgery; POAG, primary open-angle (high-tension) glaucoma; PXG, pseudoexfoliative glaucoma; trab, trabeculectomy with adjuvant MMC.

A sensitivity analysis was performed to investigate associations between survival and potential covariates including age, gender, surgery type, additional phacoemulsification surgery and preoperative IOP (table 2). Of these variables, only preoperative IOP was associated with the outcome of complete success (p=0.043). This association was quantified by a 9% higher likelihood of requiring topical therapy during the first 12 months postoperatively, for every 1 mm Hg higher preoperative IOP.

**Table 2:** Showing multivariate hazard ratios of the likelihood a patient with a given risk factor failed the criteria for complete success and qualified success after trabeculectomy surgery versus reference group

Parameter		HR associated with failure to achieve primary outcome			
		Complete success		Qualified success	
		Hazard ratio (95% CI)	P value	Hazard ratio (95% CI)	P value
**Bevacizumab**	No (control)	1.0		1.0	
	Yes	0.19 (0.05–0.71)*	0.021	0.07 (0.01–0.77)*	0.030
**Gender**	Female	1.0		1.0	
	Male	2.71 (0.87–8.47)	*NS*	1.65 (0.31–8.88)	*NS*
**Surgeon**	Surgeon 1	1.0		1.0	
	Surgeon 2	2.01 (0.58–10.47)	*NS*	10.09 (0.95–106.91)	*NS*
**Operation**	Primary trabeculectomy	1.0		1.0	
	Redo-trabeculectomy	1.23 (0.14–10.48)	*NS*	2.25 (0.21–24.62)	*NS*
**Additional phaco**	No	1.0		1.0	
	Yes	1.23 (0.31–4.92)	*NS*	2.80 (0.37–21.20)	*NS*
**Age at surgery (per year older**)		0.99 (0.93–1.04)	*NS*	0.96 (0.90–1.02)	*NS*
**Preoperative IOP (per mm Hg higher**) **glaucoma risk**		1.06 (0.99–1.13)		1.09 (0.99–1.20)	
Low		1.0	*NS*	1.0	*NS*
High		0.85 (0.28–2.58)	*NS*	0.46 (0.08–2.63)	*NS*
**Glaucoma type**					
POAG/NTG		1.0		1.0	
Other		4.61 (1.39–15.29)*	0.012	2.63 (0.53–13.05)	*NS*

*p values are provided for statistically significant results (p<0.05).

IOP, intraocular pressure; NS, not significant; NTG, normal-tension glaucoma; POAG, primary open-angle (high-tension) glaucoma.

**Figure 2:**
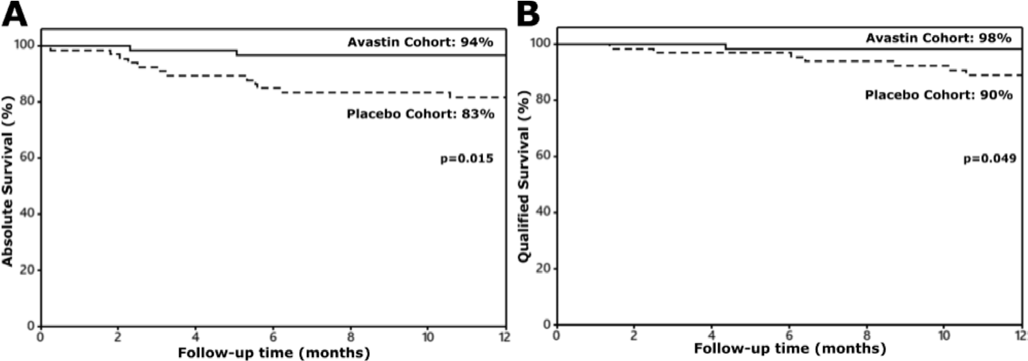
Survival curves for those who failed the criteria for complete (A) and qualified (B) success after trabeculectomy surgery stratified by randomisation allocation.

At 12 months, the proportion of patients failing to meet the criteria for ‘complete success’ was lower in the bevacizumab group (6% vs 17%; p=0.015; table 2; figure 2). Of those with POAG/NTG, 11% in the control group failed to meet the criteria for ‘complete success’ at 12 months versus 0% in the bevacizumab group (p=0.012). This association remained significant in a multivariable analysis including additional covariates of age, gender, surgery type, additional phacoemulsification surgery and preoperative IOP (p=0.014). The proportion of patients failing to meet the criteria for either ‘complete success’ or ‘qualified success’ was also lower in the bevacizumab group in a similar analysis (2% vs 10%; p=0.049). Again, among those with POAG/NTG, significantly more patients in the control group failed to meet the criteria for either ‘complete success’ or ‘qualified success’ (8%) compared with the bevacizumab group (0%, p=0.031).

**Figure 3:**
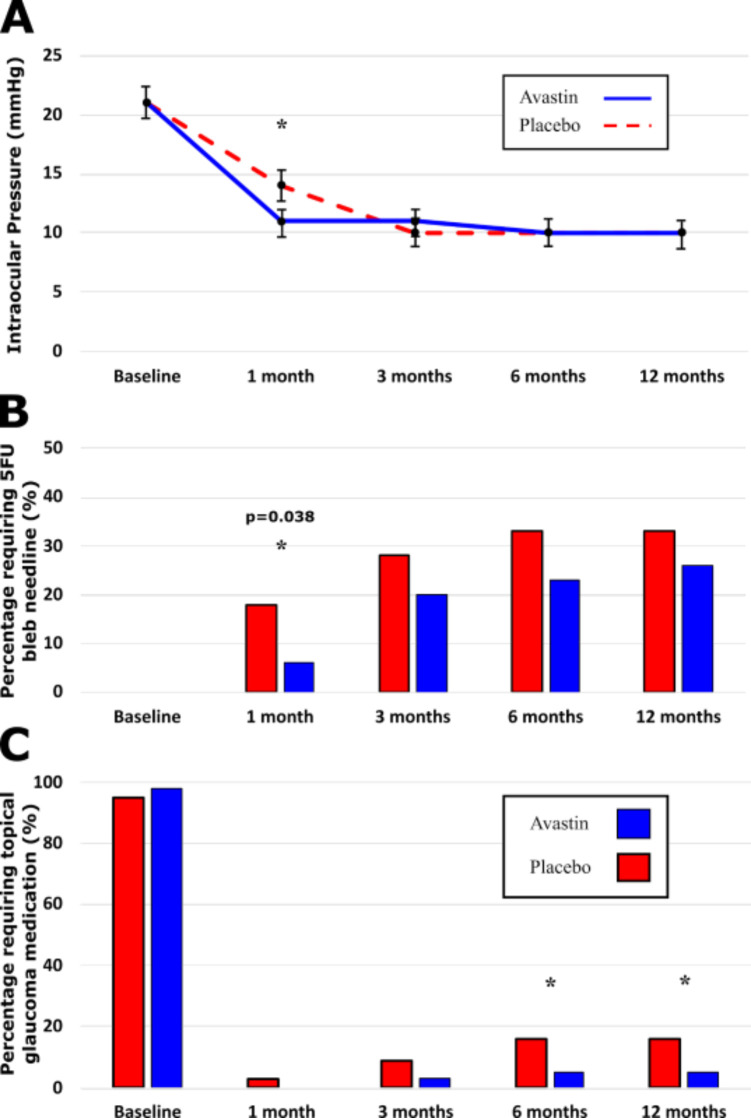
Showing: (A) Mean intraocular pressure, stratified by randomisation allocation for each time point. (B) Proportion of those requiring bleb needling with 5FU, stratified by randomisation allocation for each time point. (C) Proportion of those requiring any topical medication, stratified by randomisation allocation for each time point. 5FU, 5-fluorouracil; *p<0.05.

At baseline, there were no proportional differences between groups in the prevalence of patients requiring topical medical therapy (figure 3). At 1-month postoperatively, IOP reduction from baseline was significantly greater for the bevacizumab group (10 mm Hg vs 7 mm Hg; p=0.036). However, from 3 months postoperatively, there were no significant differences in IOP between groups. Although the required numbers of bleb needlings were nominally higher at all time points, this difference was only significant only at the 1 month time point (p=0.032). In addition, though the requirement of topical therapy was nominally higher in the control group at all time points, this association was only statistically significant from 6 months onward (6 months: p=0.047; 12 months: p=0.047).

**Figure 4:**
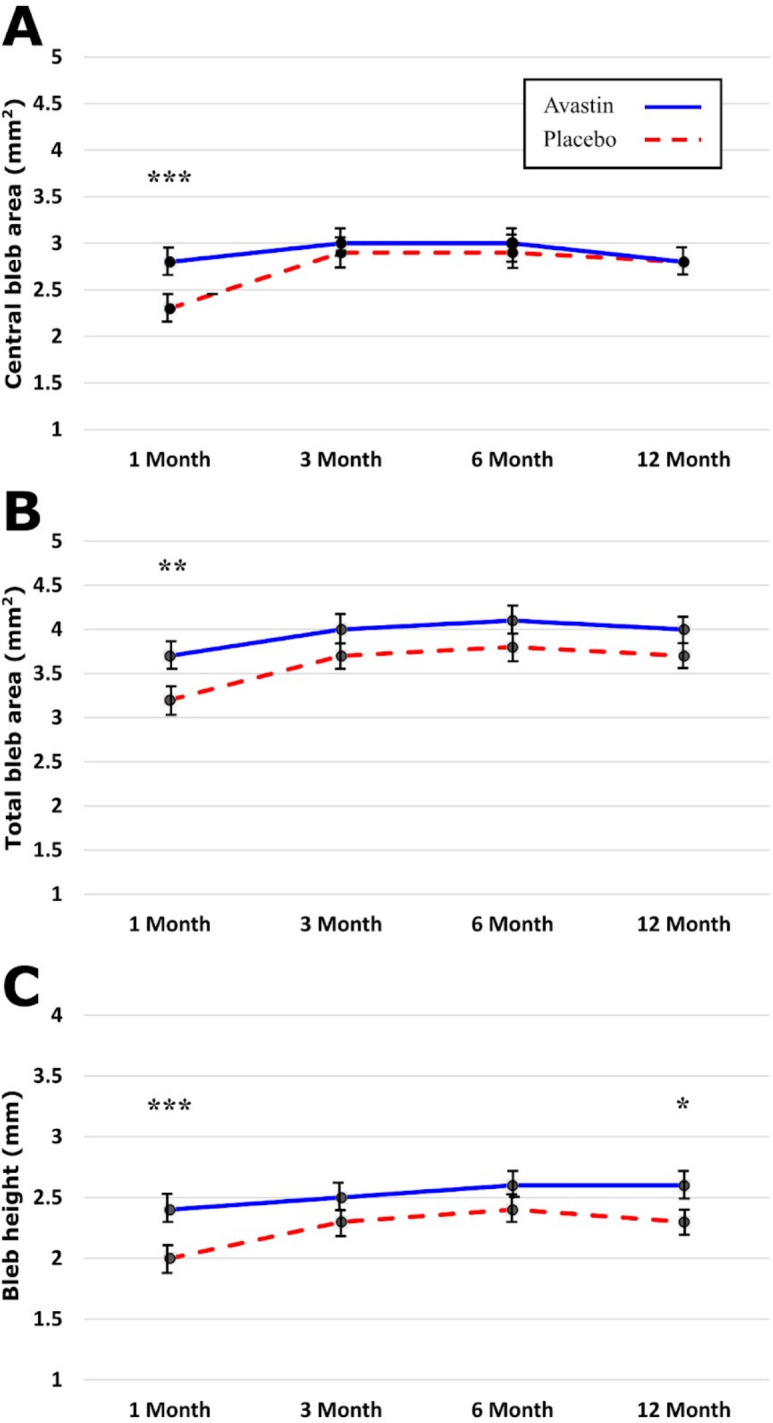
Showing bleb diffusion area: (A) central bleb area, (B) total bleb area and (C) bleb height, stratified by randomisation allocation for each time point. *p<0.05; **p<0.01; ***p<0.001.

**Figure 5:**
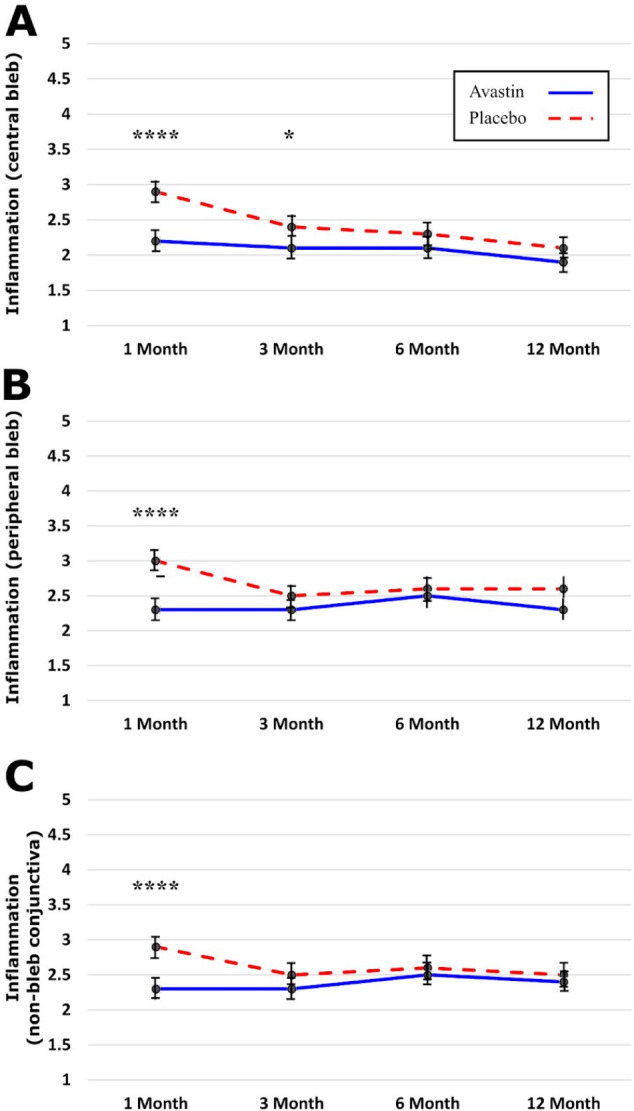
Showing vessel inflammation of bleb: (A) central bleb, (B) peripheral bleb and (C) non-bleb conjunctiva, stratified by randomisation allocation for each time point. *p<0.05; ****p<0.0001.

**Figure 6:**
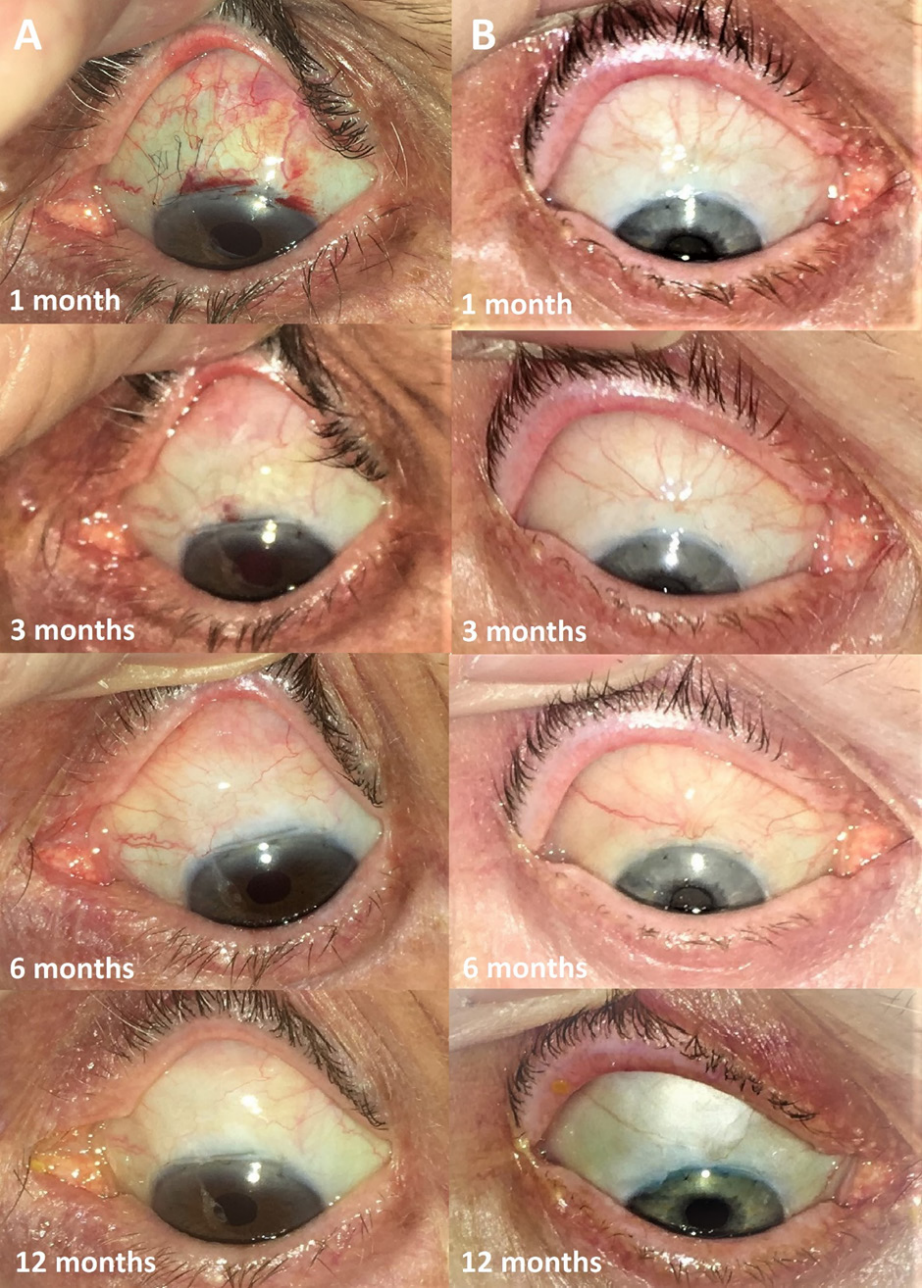
Showing bleb photos for the 1 month, 3 month, 6 month and 12 month time points, from sample patients: (A) patient 804 who received placebo and (B) patient 752, who received bevacizumab.

At 1 month, blebs in the bevacizumab group were larger in central extent (p<0.001), total extent (p<0.01) and height (p<0.001) at 1 month (figure 4). These associations were not apparent after 1 month, although bleb height remained greater in the bevacizumab group at 12 months (p=0.045). Vessel inflammation in the bevacizumab group was lower in the central bleb (p<0.0001), peripheral bleb (p<0.0001) and non-bleb conjunctiva (p<0.0001) at the 1 month time point (figure 5). Vessel inflammation subsequently reduced in the control group from 3 months, after which no differences were evident between groups (figure 6).

**Figure 7:**
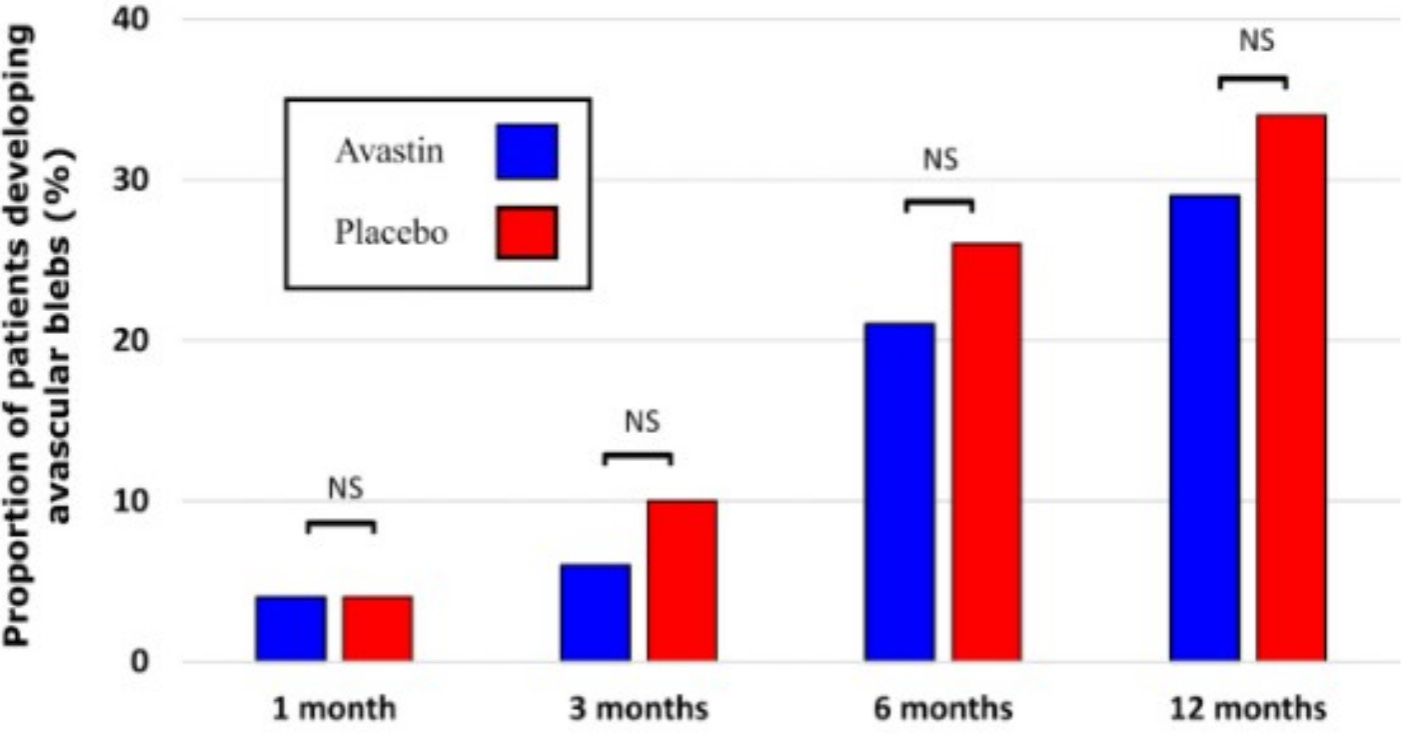
Showing proportion of those developing avascular blebs, stratified by randomisation allocation for each time point. NS, not significant.

Postoperative BCVA did not change over the first 12 months and did not differ between groups (1 month p=0.41; 3 months p=0.61; 6 months p=0.13; 12 months; p=0.37). There were no differences in the incidence of hypotonous maculopathy (p=0.65) nor the requirement for surgery to manage hypotony (p=0.40). The prevalence of avascular blebs was nominally yet non-significantly higher in the bevacizumab group at months 3, 6 and 12 (figure 7). Likewise, incident Tenon’s cysts were nominally yet non-significantly more common in the bevacizumab group (9.6% vs 1.8%; p=0.08; table 3).

**Table 3:** Showing postoperative complications stratified for randomisation allocation

Complication	Treatment group		P value
	No bevacizumab	bevacizumab	
Early postoperative period (within 1 month)			
Central retinal vein occlusion	1 (1.8%)	0 (0.0%)	*NA*
Hypotonous maculopathy	2 (3.6%)	2 (3.8%)	0.91
Choroidal effusion	1 (1.8%)	3 (5.8%)	0.27
Hyphema	2 (3.6%)	1 (1.9%)	0.58
Wound leak	1 (1.8%)	2 (3.8%)	0.47
Late postoperative period (from 1 month to 12 months)			
Blebitis/endophthalmitis	1 (1.8%)	0 (0.0%)	*NA*
Tenon’s cyst	1 (1.8%)	5 (9.6%)	0.08
Microbial keratitis	1 (1.8%)	1 (1.9%)	0.89
Corneal endothelial failure	1 (1.8%)	0 (0.0%)	*NA*
Hypotonous maculopathy	2 (3.6%)	3 (5.8%)	0.66

Although no vision threatening complications were observed within the bevacizumab group, one central retinal vein obstruction and one blebitis/endophthalmitis occurred in the control group. There were no differences in any non-vision-threatening complications between groups (table 3).

## Discussion

As the favoured surgical intervention in glaucoma, trabeculectomy has a proven track record of success, having been performed now for over 50 years. However, improvements can always be made to any management strategy and although, given its high rates of success generally, any changes are only likely to lead to incremental improvements, if these changes are simple, low cost and come with minimal morbidity, then a change in practice may be warranted.

We demonstrated that, when combined with conventional MMC augmentation, a single intraoperative adjunctive intravitreal bevacizumab injection resulted in a lower IOP during the first month compared with MMC alone (the control group). In subsequent months, similar IOP between the control and the intervention groups was only able to be achieved because of a higher rate of bleb needling with 5-FU at the 3-month time point and later due to the addition of topical therapy (figure 3).

Only 6% of those who received intravitreal bevacizumab required supplemental topical medication to achieve the target IOP at the end of the first year (complete success) compared with 17% of control patients. In addition, only 2% of bevacizumab patients required further glaucoma surgery compared with 10% of control patients (qualified success; figure 2) during the same period. Morphologically, trabeculectomy blebs, among the bevacizumab group, were of larger volume in central extent, total extent and height and had less prominent vessel inflammation over the central bleb, the peripheral bleb and the non-bleb conjunctiva at 1 month postoperatively (figures 4 and 5). This time point might correspond with the time during which bevacizumab is maximally effective.^12^ In latter months, there was also a decrease in vessel inflammation within the blebs of the control group as well as an increase in their volume, due to a greater number of bleb needlings. This resulted in the blebs from the control and the bevacizumab group becoming statistically similar by the 12-month time point.

Bevacizumab interrupts the fibroproliferative phase of wound healing. The effect of this would be less thickening and, thus, less resistance to aqueous diffusion at the level of the tenon’s capsule and the conjunctiva. Previous work has raised concerns that bevacizumab might lead to a higher prevalence of avascular blebs or tenon’s cysts.^28 29^ Our paper showed that although the prevalence of avascular blebs increased over the 12-month follow-up time, it did so in both groups with no significant differences. In addition, there was a trend towards more tenon’s cysts among the bevacizumab groups; however, this did not reach statistical significance.

Although trabeculectomies in the bevacizumab group led to lower IOP at the 1 month time point compared with the control group, this did not result in a significantly higher rate of hypotony (p=0.66), nor a higher rate of return to theatre to treat hypotony (p=0.40; table 3). There were a higher number of vision-threatening complications among the control group, although this small number did not reach statistical significance. However, there were no statistical differences between the groups in relation to non-vision-threatening complications. Although rare complications associated with intravitreal injections (including lens touch, vitreous haemorrhage, or retinal detachment) were not seen in the current study, these are relevant considerations when considering adjuvant administration of intravitreal anti-VEGF therapies in trabeculectomy+MMC surgery.

This is the first paper to be able to demonstrate a positive effect of bevacizumab on the outcomes of trabeculectomy surgery. Previous papers examining the use of intraoperative anti-VEGF during trabeculectomy surgery in addition to MMC have found no effect on survival time (table 4).^30–33^ However, there has only been one paper which used an intravitreal delivery for the drug.^30^ Two other papers have given the anti-VEGF agent subconjunctivally (SC)^32 33^ and one has given it intracamerally (IC).^31^ However, drugs delivered to these latter two locations are likely to lose effectiveness within 14 days (SC) to 18 days (IC),^34 35^ compared with 100 days for intravitreal drug delivery.^12^ Crucially, however, all of these previous papers have been underpowered. In addition, attempts at combining results of some of these papers in a meta-analysis failed to yield any positive results.^29 36^

**Table 4:** Summary of previous randomised controlled studies investigating the effects of adjuvant anti-VEGF on success following trabeculectomy with MMC

Study	Anti-VEGF agent	Location of administration	Follow-up time (months)	Treatment group (n)		Events (% of total)		Complete success (% of total)	
				Treatment	Control	Treatment	Control	Treatment	Control
Kahook, M.Y.	Ranibizumab (Lucentis)	Intravitreal	6	5	5	0 (0%)	0 (0%)	100%	100%
Vanderwalle, E. *et al*	Bevacizumab (Avastin)	Intracameral	12	32	34	8 (25%)	16 (47%)	75% (71%)	53% (51%)
Saeed, A.M. *et al*	Bevacizumab (Avastin)	Subconjunctival	24	13	13	3 (23%)	5 (38%)	77%	54%
Kiddee, W. *et al*	Bevacizumab (Avastin)	Subconjunctival	12	20	19	9 (45%)	8 (42%)	55%	58%

MMC, mitomycin-C; VEGF, vascular endothelial growth factor.

Despite randomisation, several cohort differences were identified in the final analysis. Primarily, the prevalence of combined phacoemulsification-trabeculectomy was higher in the bevacizumab group (33% vs 18%; p=0.04). As no difference in postoperative complication rates was observed in a single RCT comparing trabeculectomy with combined phacoemulsification-trabeculectomy, this observation is unlikely to have affected our results.^37^ Secondarily, our control group included five cases of uveitic glaucoma while the bevacizumab group had none. A retrospective review of these cases revealed that four resulted in outcome of ‘complete success’, and one failed due to a requirement for recommencement of topical therapy at 1 week, and progression to redo-trabeculectomy at 1 month. Notably, neither treating uveitic glaucoma as a covariate nor exclusion of uveitic glaucoma cases from the analysis significantly affected the primary outcome.

The limitations of this study include the short follow-up time. Adverse surgical outcomes including bleb avascularity and bleb failure may occur many years postoperatively. However, from previous work, we can see that most trabeculectomy failures occur during the first 12 months, with the most rapid period of failure being the first 2 months. Our study encompassed this critical period. The strengths of our study include a double-blinded design for a surgical study. In addition, we have powered our study two times, which has been performed previously. This study was also limited by its inability to account for combined phacoemulsification trabeculectomy as a potential randomisation factor. Because of this, randomisation resulted in different proportions of combined surgery between groups, a factor which could have resulted in confounding. However, we do not anticipate that combined surgery would have impacted the current study’s results, as previous studies have demonstrated combined phacotrabeculectomy+MMC to result in either similar,^38^ or worse rates of surgical success^39 40^ and either similar or lesser IOP-lowering effects.^39 41^ Based on these results, greater randomisation of phacotrabeculectomy+MMC to the bevacizumab group should have resulted in greater success rates in the control group.

In conclusion, our study has demonstrated that intravitreal adjunctive bevacizumab given intraoperatively during trabeculectomy results in a significant reduction in the need for additional medication and further surgery to achieve target IOP. In addition, it results in larger and less inflamed blebs, which require less interventions, while not producing a significant increase in hypotony or bleb avascularity. Future work should seek to replicate these results and investigate which subgroups may benefit most from this intervention.

## Data Availability

No data are available.

## References

[1] Rodriguez-Una I , Azuara-Blanco A , King AJ . Survey of glaucoma surgical preferences and post-operative care in the United Kingdom. Clin Exp Ophthalmol 2017;45:232–40. 10.1111/ceo.12846 27726283

[2] Goldenfeld M , Krupin T , Ruderman JM , et al . 5-fluorouracil in initial Trabeculectomy. A prospective, randomized, multicenter study. Ophthalmology 1994;101:1024–9. 10.1016/s0161-6420(94)31223-1 8008342

[3] De Fendi LI , Arruda GV , Scott IU , et al . Mitomycin C versus 5-fluorouracil as an adjunctive treatment for Trabeculectomy: a meta-analysis of randomized clinical trials. Clin Exp Ophthalmol 2013;41:798–806. 10.1111/ceo.12097 24308066

[4] Wilkins M , Indar A , Wormald R . Intra-operative mitomycin C for glaucoma surgery. Cochrane Database Syst Rev 2005;2005:CD002897. 10.1002/14651858.CD002897.pub2 16235305 PMC8406713

[5] Skuta GL , Parrish RK . Wound healing in glaucoma filtering surgery. Surv Ophthalmol 1987;32:149–70. 10.1016/0039-6257(87)90091-9 3328315

[6] Bates DO , Hillman NJ , Williams B , et al . Regulation of Microvascular permeability by vascular endothelial growth factors. J Anat 2002;200:581–97. 10.1046/j.1469-7580.2002.00066.x 12162726 PMC1570751

[7] Lopilly Park H-Y , Kim JH , Ahn MD , et al . Level of vascular endothelial growth factor in tenon tissue and results of glaucoma surgery [Internet]. Arch Ophthalmol 2012;130:685–9. 10.1001/archophthalmol.2011.2799 22332204

[8] Li Z , Van Bergen T , Van de Veire S , et al . Inhibition of vascular endothelial growth factor reduces scar formation after glaucoma filtration surgery. Invest Ophthalmol Vis Sci 2009;50:5217–25. 10.1167/iovs.08-2662 19474408

[9] Kazazi-Hyseni F , Beijnen JH , Schellens JHM . Bevacizumab. Oncologist 2010;15:819–25. 10.1634/theoncologist.2009-0317 20688807 PMC3228024

[10] Penn JS , Madan A , Caldwell RB , et al . Vascular endothelial growth factor in eye disease. Prog Retin Eye Res 2008;27:331–71. 10.1016/j.preteyeres.2008.05.001 18653375 PMC3682685

[11] Vandewalle E , Van de S , et al . The role of different VEGF Isoforms in scar formation after glaucoma filtration surgery. Exp Eye Res 2011:689–99. 21907194 10.1016/j.exer.2011.08.016

[12] Shah AR , Del Priore LV . Duration of action of intravitreal Ranibizumab and Bevacizumab in Exudative AMD eyes based on macular volume measurements. Br J Ophthalmol 2009;93:1027–32. 10.1136/bjo.2008.149674 19429594

[13] Coote MA , Ruddle JB , Qin Q , et al . Vascular changes after intra-Bleb injection of Bevacizumab. J Glaucoma 2008;17:517–8. 10.1097/IJG.0b013e31815f5345 18854726

[14] Grewal DS , Jain R , Kumar H , et al . Evaluation of subconjunctival Bevacizumab as an adjunct to Trabeculectomy a pilot study. Ophthalmology 2008;115:2141–5. 10.1016/j.ophtha.2008.06.009 18692246

[15] Landers J , Martin K , Sarkies N , et al . A twenty-year follow-up study of Trabeculectomy: risk factors and outcomes. Ophthalmology 2012;119:694–702. 10.1016/j.ophtha.2011.09.043 22196977

[16] Musch DC , Lichter PR , Guire KE , et al . The collaborative initial glaucoma treatment study: study design, methods, and baseline characteristics of enrolled patients. Ophthalmology 1999;106:653–62. 10.1016/s0161-6420(99)90147-1 10201583

[17] Shaarawy TM , Sherwood MB , Grehn F . Guidelines on Design and Reporting of Glaucoma Surgical Trials. Kugler Publications, 2009: 83.

[18] Wells AP , Crowston JG , Marks J , et al . A pilot study of a system for grading of drainage blebs after glaucoma surgery. J Glaucoma 2004;13:454–60. 10.1097/00061198-200412000-00005 15534469

[19] Casson R , Rahman R , Salmon JF . Long term results and complications of Trabeculectomy augmented with low dose mitomycin C in patients at risk for filtration failure. Br J Ophthalmol 2001;85:686–8. 10.1136/bjo.85.6.686 11371489 PMC1724022

[20] Robinson DI , Lertsumitkul S , Billson FA , et al . Long-term intraocular pressure control by Trabeculectomy: a ten-year life table [Internet]. Aust N Z J Ophthalmol 1993;21:79–85. 10.1111/j.1442-9071.1993.tb00758.x 8101448

[21] Ehrnrooth P , Lehto I , Puska P , et al . Long-term outcome of Trabeculectomy in terms of intraocular pressure. Acta Ophthalmol Scand 2002;80:267–71. 10.1034/j.1600-0420.2002.800307.x 12059864

[22] Diestelhorst M , Khalili MA , Krieglstein GK . Trabeculectomy: a retrospective follow-up of 700 eyes. Int Ophthalmol 1998;22:211–20. 10.1023/a:1006238624072 10674865

[23] Edmunds B , Thompson JR , Salmon JF , et al . The National survey of Trabeculectomy. II. Variations in Operative Technique and Outcome Eye 2001:441–8. 10.1038/eye.2001.152 11767016

[24] Parc CE , Johnson DH , Oliver JE , et al . The long-term outcome of glaucoma filtration surgery. Am J Ophthalmol 2001;132:27–35. 10.1016/s0002-9394(01)00923-0 11438050

[25] Prata Júnior JA , Minckler DS , Baerveldt G , et al . Trabeculectomy in pseudophakic patients: postoperative 5-fluorouracil versus intraoperative mitomycin C antiproliferative therapy. Ophthalmic Surg 1995;26:73–7. 7746631

[26] Scott IU , Greenfield DS , Schiffman J , et al . Outcomes of primary Trabeculectomy with the use of adjunctive mitomycin. Arch Ophthalmol 1998;116:286–91. 10.1001/archopht.116.3.286 9514480

[27] Gedde SJ , Feuer WJ , Lim KS , et al . Treatment outcomes in the primary tube versus Trabeculectomy study after 3 years of follow-up. Ophthalmology 2020;127:333–45. 10.1016/j.ophtha.2019.10.002 31727428

[28] Chua BE , Nguyen DQ , Qin Q , et al . Bleb Vascularity following post-Trabeculectomy subconjunctival bevacizumab: a pilot study. Clin Exp Ophthalmol 2012;40:773–9. 10.1111/j.1442-9071.2012.02798.x 22429268

[29] Liu X , Du L , Li N . The effects of Bevacizumab in augmenting Trabeculectomy for glaucoma: a systematic review and meta-analysis of randomized controlled trials. Medicine (Baltimore) 2016;95:e3223. 10.1097/MD.0000000000003223 27082560 PMC4839804

[30] Kahook MY . Bleb morphology and vascularity after Trabeculectomy with intravitreal ranibizumab: a pilot study. Am J Ophthalmol 2010;150:399–403. 10.1016/j.ajo.2010.03.025 20570237

[31] Vandewalle E , Abegão Pinto L , Van Bergen T , et al . Intracameral bevacizumab as an adjunct to Trabeculectomy: a 1-year prospective, randomised study. Br J Ophthalmol 2014;98:73–8. 10.1136/bjophthalmol-2013-303966 24158846

[32] Saeed AM , AboulNasr TT . Subconjunctival Bevacizumab to augment Trabeculectomy with mitomycin C in the management of failed glaucoma surgery. Clin Ophthalmol 2014;8:1745–55. 10.2147/OPTH.S67730 25246758 PMC4168860

[33] Kiddee W , Orapiriyakul L , Kittigoonpaisan K , et al . Efficacy of adjunctive subconjunctival bevacizumab on the outcomes of primary Trabeculectomy with mitomycin C: A prospective randomized placebo-controlled trial. J Glaucoma 2015;24:600–6. 10.1097/IJG.0000000000000194 25393038 PMC4614532

[34] Chen W-L , Lin C-T , Lin N-T , et al . Subconjunctival injection of Bevacizumab (Avastin) on corneal neovascularization in different rabbit models of corneal angiogenesis. Invest Ophthalmol Vis Sci 2009;50:1659–65. 10.1167/iovs.08-1997 18997093

[35] Wolf A , von Jagow B , Ulbig M , et al . Intracameral injection of Bevacizumab for the treatment of Neovascular glaucoma. Ophthalmologica 2011;226:51–6. 10.1159/000327364 21546781

[36] Chen HJ , Lin C , Lee CH , et al . Efficacy and safety of bevacizumab combined with mitomycin C or 5-fluorouracil in primary Trabeculectomy: a meta-analysis of randomized clinical trials. Ophthalmic Res 2018;59:155–63. 10.1159/000486576 29533959

[37] Wang M , Fang M , Bai Y , et al . Comparison of combined Phacotrabeculectomy with Trabeculectomy only in the treatment of primary angle-closure glaucoma. Chin Med J (Engl) 2012;125:1429–33. 22613648

[38] Murthy SK , Damji KF , Pan Y , et al . Trabeculectomy and Phacotrabeculectomy, with mitomycin-C, show similar two-year target IOP outcomes. Can J Ophthalmol 2006;41:51–9. 10.1016/S0008-4182(06)80067-0 16462873

[39] Sacchi M , Monsellato G , Villani E , et al . Intraocular pressure control after combined Phacotrabeculectomy versus Trabeculectomy alone. Eur J Ophthalmol 2022;32:327–35. 10.1177/1120672121999997 33685259

[40] Ogata-Iwao M , Inatani M , Takihara Y , et al . A prospective comparison between Trabeculectomy with mitomycin C and Phacotrabeculectomy with mitomycin C. Acta Ophthalmol 2013;91:e500–1. 10.1111/aos.12133 23617934

[41] E Graf N , Müller M , Gerlach F , et al . Comparison of 2-year-results of mitomycin C-augmented Trabeculectomy with or without cataract extraction in glaucoma patients. Can J Ophthalmol 2019;54:347–54. 10.1016/j.jcjo.2018.07.006 31109475

